# Coiled-coil domain containing 50-V2 protein positively regulates neurite outgrowth

**DOI:** 10.1038/s41598-020-78304-3

**Published:** 2020-12-04

**Authors:** Ju-Sik Min, Debasish Halder, Ji-Yong Yoon, Su-Jin Jeon, Soo Young Jun, Jae-Ran Lee, Jeong-Ju Lee, Min-Hyuk Choi, Cho-Rok Jung, DaYong Lee, Byoung-Joon Kim, Nam-Soon Kim

**Affiliations:** 1 Rare Disease Research Center, Korea Research Institute of Bioscience and Biotechnology (KRIBB), Daejeon, 34141 Republic of Korea; 2 Genome Research Center, Korea Research Institute of Bioscience and Biotechnology, Daejeon, 34141 Republic of Korea; 3 Department of Functional Genomics, Korea University of Science and Technology, Daejeon, 34113 Republic of Korea; 4 Department of Neurology, Sungkyunkwan University School of Medicine, Samsung Medical Center, Gangnam-gu, Seoul, 06351 Republic of Korea; 5 New Drug Development Center, Daegu-Gyeongbuk Medical Innovation Foundation, Daegu, Republic of Korea

**Keywords:** Neuroscience, Molecular biology

## Abstract

The coiled-coil domain containing 50 (CCDC50) protein is a phosphotyrosine-dependent signalling protein stimulated by epidermal growth factor. It is highly expressed in neuronal cells in the central nervous system; however, the roles of CCDC50 in neuronal development are largely unknown. In this study, we showed that the depletion of CCDC50-V2 impeded the neuronal development process, including arbor formation, spine density development, and axonal outgrowth, in primary neurons. Mechanistic studies revealed that CCDC50-V2 positively regulated the nerve growth factor receptor, while it downregulated the epidermal growth factor receptor pathway. Importantly, JNK/c-Jun activation was found to be induced by the CCDC50-V2 overexpression, in which the interaction between CCDC50-V2 and JNK2 was also observed. Overall, the present study demonstrates a novel mechanism of CCDC50 function in neuronal development and provides new insight into the link between CCDC50 function and the aetiology of neurological disorders.

## Introduction

The coiled-coil domain-containing 50 (CCDC50) protein (also termed Ymer) was identified as a phosphotyrosine-dependent signalling protein in epidermal growth factor (EGF)-stimulated human epidermoid carcinoma cells^1^. CCDC50 is a member of the A20/Tnfaip3 ubiquitin-editing complex and is involved in EGF-receptor-mediated deubiquitination for the inhibition of the downregulation of the EGF receptor (EGFR)^2–4^. This protein acts as a multifunctional regulator of inflammation, cell death, and proliferation^5,6^. CCDC50 is highly expressed in neuronal cells of the central nervous system (CNS) as well as in immune cells of the bone marrow, spleen, appendix, and lymph nodes^7–11^. CCDC50 was shown to be spatiotemporally distributed with the microtubule-based cytoskeleton in hair cells of the organ of Corti, a receptor organ responsible for hearing. Previously, it was demonstrated that the loss of CCDC50 function results in the disorganization of the microtubule-based cytoskeleton in hair cells and causes progressive hearing loss in autosomal dominant deafness 44 (DFN44)^3^. Although CCDC50 is dynamically expressed during inner ear development, in adults, it is prominently associated with cochlear hair cells, the functional impairment of which leads to alterations in intracortical network activities^3,12,13^. This suggests that CCDC50 may have an imperative role in neuronal development, but direct evidence has not yet been reported.

Two isoforms of the human *CCDC50* gene were previously reported. Human *CCDC50* variant 1 (*CCDC50-V1*, NM_174908.3) contains 306 amino acids (921 bp CDS) with skipping of exon 6, whereas human *CCDC50* variant 2 (*CCDC50-V2*, NM_178335.2) consists of 482 amino acids (1449 bp CDS)^14^. In addition, mouse *Ccdc50* variant 1 (*Ccdc50-V1*, NM_026202.2) lacks exon 6, corresponds to human *CCDC50-V1* and encodes a protein of 305 amino acids. Mouse *Ccdc50* variant Vx3 (*Ccdc50-Vx3*, XM_011246002.3) corresponds to human *CCDC50-V2* was predicted to encode a protein of 480 amino acids (1443 bp CDS). To date, the functional role of human CCDC50-V2 (Ccdc50-Vx3 in mouse), especially in regulating neuronal phenotypes, has yet to be determined.

The complex array of specific cellular responses that relay signals from canonical systems is known to govern cell fate. Two growth hormones, EGF and nerve growth factor (NGF), interact with the receptor tyrosine kinases (RTKs) and stimulate the signalling cascades to induce cell proliferation and differentiation, respectively. Therefore, the signalling molecules provide cues to modulate the activation of the canonical signalling cassette (i.e., ERK) and drive either proliferation or differentiation^8,9^. Consequently, a closely related RTK that is activated by NGF, nerve growth factor receptor (NGFR) (also known as p75^NTR^) and TrkA receptor tyrosine kinase, induces neuronal differentiation by stopping the proliferation of cells. The NGFR signalling pathway is a representative growth factor/receptor pathway with intrinsic tyrosine-kinase activity that promotes structural changes and the axonal growth of neuronal cells^7^. NGFR, in response to NGF, induces the phosphorylation of JNK isoforms in hippocampal and granular neuron cells in the developing brain^15^. In addition, in response to sustained activation of ERK, NGF recruits the Shc-Grb2-Sos complex to the TrkA receptor, thereby activating several downstream protein signalling pathways, including the phosphatidylinositol 3-kinase (PI3K) pathway. PI3K then activates JNK, which, through the activation of c-Jun, can promote differentiation^9,16,17^.

In this study, we uncovered a new mechanism of CCDC50-V2 function in neuronal development. Using multiple types of cultured neurons, we demonstrated that CCDC50-V2 controls neuronal development by regulating axonal guidance, especially during arbor formation, spine density and axonal growth. Moreover, CCDC50-V2 induces the JNK/c-Jun activation and the positive regulation of NGFR expression, while it negatively regulates the EGFR pathway, thereby playing a crucial role in cell fate control during neural development. These findings support an important role for CCDC50-V2 in neuronal development through the regulation of signalling pathways associated with cell fate control and neuronal differentiation.

## Results

### *CCDC50-V2* knockdown inhibits axon and dendrite outgrowth

**Figure 1:**
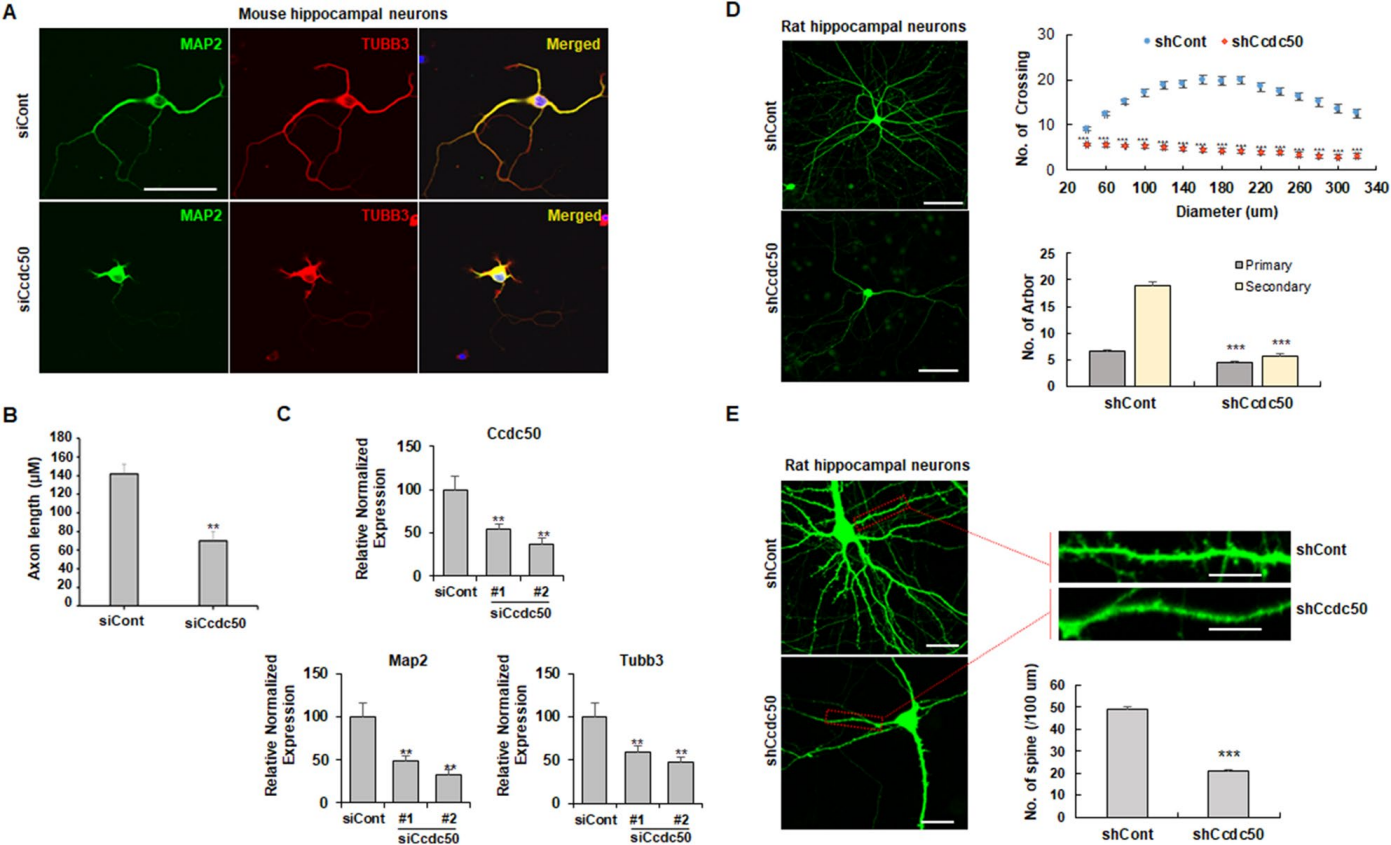
*CCDC50* knockdown suppresses dendritic and axonal outgrowth in primary cultured neurons. (A-C) Mouse primary hippocampal neurons were transiently transfected with control siRNA (siCont) and siRNAs against the murine *CCDC50* gene on day in vitro 3 (DIV 3). (**A**) After 72 h of incubation, the cells were stained for MAP2 (green) and TUBB3 (red) (**A**). Scale bars = 30 µm. (**B**) Axonal lengths were measured and are presented as the proportion of the dendritic length of TUBB3-positive and MAP2-negative dendrites. Over 40 cells were measured in three independent experiments. (**C**) The expression levels of neuronal markers such as *Map2* and *Tubb3* in primary neurons treated with two siRNAs against *Ccdc50* (siCcdc50# 1 and siCcdc50# 2) were analysed by quantitative real time RT-PCR. Data presented were normalized to Gapdh. (D, E) Analysis of the attenuation of dendritic arbor development and spine density in *Ccdc50* knockdown neuronal cells. Rat primary hippocampal neurons were stably transfected with green fluorescent protein (GFP)-tagged control (shCont) or shCcdc50 (a vector that silences all variants of rat *Ccdc50*) on DIV 5; then, immunostaining with an anti-EGFP antibody was performed on DIV 9 (**D**; left panel). The quantification of dendrite complexity by Sholl analysis of rat primary hippocampal neurons (**D**; upper right). The numbers of primary and secondary dendrites were lower in *Ccdc50* knockdown cells than in control cells (**D**; lower right). Means ± SD of data from 30 control neurons and 30 CCDC50 knock down neurons are shown. (**E**) The densities of the dendritic spines were compared between control (shCont)- and shCcdc50-treated cells. Means ± SD of data from 84 dendrites for control and 79 dendrites for CCDC50 knock down are shown. *p < 0.05, **p < 0.01, ***p < 0.001, Student’s t-test compared with the control group.

To investigate the role of CCDC50 in neuronal development, we first examined the suppressive effect of CCDC50 on the development of neuronal phenotypes. Mouse primary hippocampal neurons were treated with small interfering RNA (siRNA) against *CCDC50,* and the axonal and dendritic growth patterns of the transfected neurons were examined using immunocytochemistry and quantitative real time RT-PCR analysis. We observed a significant decrease in axon length in Ccdc50 knockdown mouse hippocampal neurons (Fig. 1A,B). The results also showed that compared to that in the control group, the silencing of *Ccdc50* markedly decreased the expression of neuronal markers such as *Map2* and *Tubb3* (Fig. 1C). In addition, *Ccdc50* depletion in rat hippocampal neurons significantly reduced spine density as well as dendritic arborization, impeding the formation of secondary dendrites (Fig. 1D,E). These observations suggest that normal function of CCDC50 is necessary for the outgrowth of neurites.

**Figure 2:**
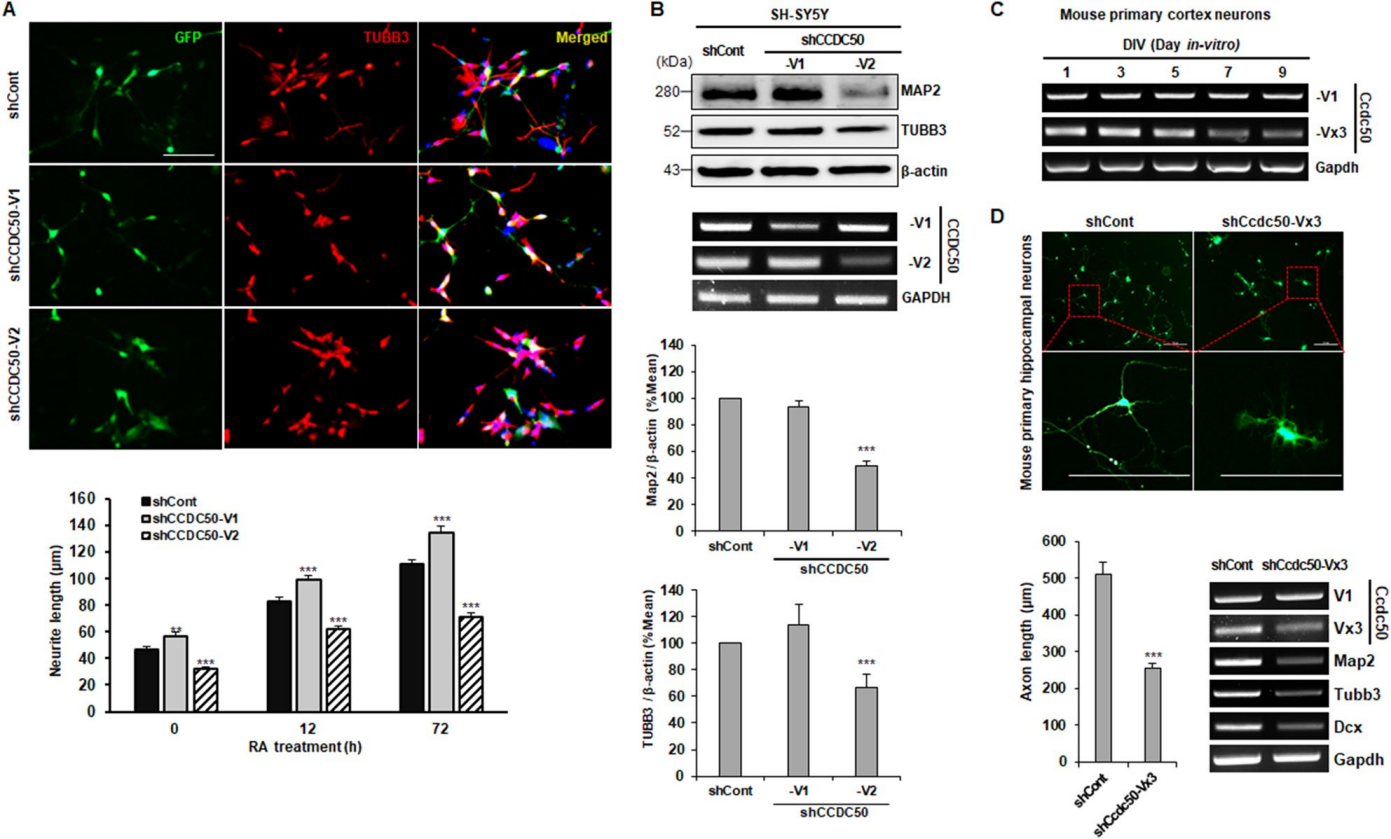
*CCDC50-V2* knockdown decreases neuronal differentiation. (**A**) Human SH-SY5Y neuroblastoma cells were transfected with shCont, shCCDC50-V1 (human CCDC50-V1) and shCCDC50-V2 (human CCDC50-V2) and then treated with retinoic acid (RA, 10 µM) for the indicated time periods. The cells were immunostained with anti-EGFP (green), anti-TUBB3 (red, neuron-specific actin cytoskeleton), and DAPI (blue, nuclei). Scale bar = 100 µm. Neurite lengths were measured in approximately 30 GFP-expressing cells and compared in three independent experiments (**A**; lower panel) and graphically presented. (**B**) The expression levels of MAP2 and TUBB3 in transfected cells were measured using western blotting (upper panels) and semi-quantitative RT-PCR analysis (lower panels). (**C**) The mRNA expression patterns of *Ccdc50* variants in mouse primary cortical neurons were analysed by semi-quantitative RT-PCR. (**D**) A reduction in neurite outgrowth was detected in mouse primary hippocampal neurons transfected with GFP-tagged shControl (shCont) or shCcdc50-Vx3 (a vector that silences the long variant of mouse CCDC50) on DIV 3. Representative images of the GFP-expressing cells were acquired on DIV 6 (upper panel; scale bar = 200 μm). The quantification of neurite outgrowth was performed using NIS Elements software, and the neurite length was measured in approximately 30 GFP-expressing cells in three independent experiments (lower left panel). The downregulation of neuronal markers in primary neurons treated with shCcdc50-Vx3 was detected by semi-quantitative RT-PCR (lower right panel). **p < 0.01, ***p < 0.001, Student’s t-test compared with the control group.

To examine the role of the two *CCDC50* variants in neuronal development, the effect of *CCDC50-V1* and *CCDC50-V2* knockdown on neuronal differentiation was evaluated. For this purpose, human SH-SY5Y neuroblastoma cells were transfected with two plasmids, shCCDC50-V1 and shCCDC50-V2, which are silencing vectors of human *CCDC50-V1* and *CCDC50-V2* (Supplemental Figure S1A), respectively. The results showed that the depletion of *CCDC50-V2* in SH-SY5Y cells led to a decrease in neurite length compared to that in the control group (Fig. 2A). In particular, under retinoic acid (RA)-stimulated neuronal differentiation for 72 h, *CCDC50-V2* silencing, but not *CCDC50-V1* silencing, inhibited neurite outgrowth in SH-SY5Y cells (Fig. 2A; lower panel). Although a decrease in neurite outgrowth is also observed in CCDC50-V2 knockdown cells before RA treatment, this decrease may result from the knockdown effects of CCDC50-V2 plasmid during the pre-incubation of undifferentiated SH-SY5Y cells with transfection reagent and plasmid. Furthermore, the expression of the neuronal markers MAP2 and TUBB3 was greatly decreased in *CCDC50-V2*-silenced cells, but very little or no inhibition of these markers occurred in *CCDC50-V1*-silenced cells (Fig. 2B). Together, these findings suggest that CCDC50-V2 might be more actively involved in neurite outgrowth process than CCDC50-V1.

Computational analysis has predicted the existence of a murine *CCDC50 long variant transcript* (*Ccdc50-Vx3*; XM_011246002.3), and that 81% of the nucleotide sequences and 82% of the amino acid sequences are similar to human CCDC50-V2 (NM_178335.2). To confirm the expression of *Ccdc50-Vx3* in mouse primary neurons, we performed RT-PCR analysis with specific primers for mouse *CCDC50* (Table S1); then the PCR product was allowed to sequence. As expected, *Ccdc50-Vx3* transcript, including exon Vx3 (amino acids 449-973), was confirmed (Supplemental Figure S1B). To further explore the role of Ccdc50-V1 and Ccdc50-Vx3 in neuronal development, we evaluated the expression patterns of the two *Ccdc50* variants at three early developmental time points in mice (Fig. 2C and Supplementary Fig. S1C). Surprisingly, *Ccdc50-Vx3,* the long variant in mice, was highly expressed in mouse primary neuronal cells until day 5, but its expression dramatically decreased afterwards. Conversely, the expression of *Ccdc50-V1* was almost unchanged through the entire time period investigated (Fig. 2C). Additionally, *Ccdc50-Vx3* protein expression was extremely low in the cerebral cortex at embryonic stages but high on postnatal day 1, when vigorous neurogenesis was activated (Supplementary Fig. S1C). Unlike *Ccdc50-V1*, *Ccdc50-Vx3* was found to be highly expressed in the brain tissues of C57BL/6J mice (Supplementary Fig. S1D)*.* Furthermore, the silencing of *Ccdc50-Vx3* decreased axonal outgrowth and the expression of neuronal markers (*Map2*, *Tubb3*, and *Dcx*) in mouse primary hippocampal neurons compared to that in the control group (Fig. 2D). Taken together, these findings suggest that *CCDC50-V2* (*Ccdc50-Vx3* in mice) is involved in axonal and dendritic outgrowth in both primary and cultured neurons as well as in neuronal development under in vivo conditions.

### CCDC50-V2 inhibits the EGFR signalling pathway

**Figure 3:**
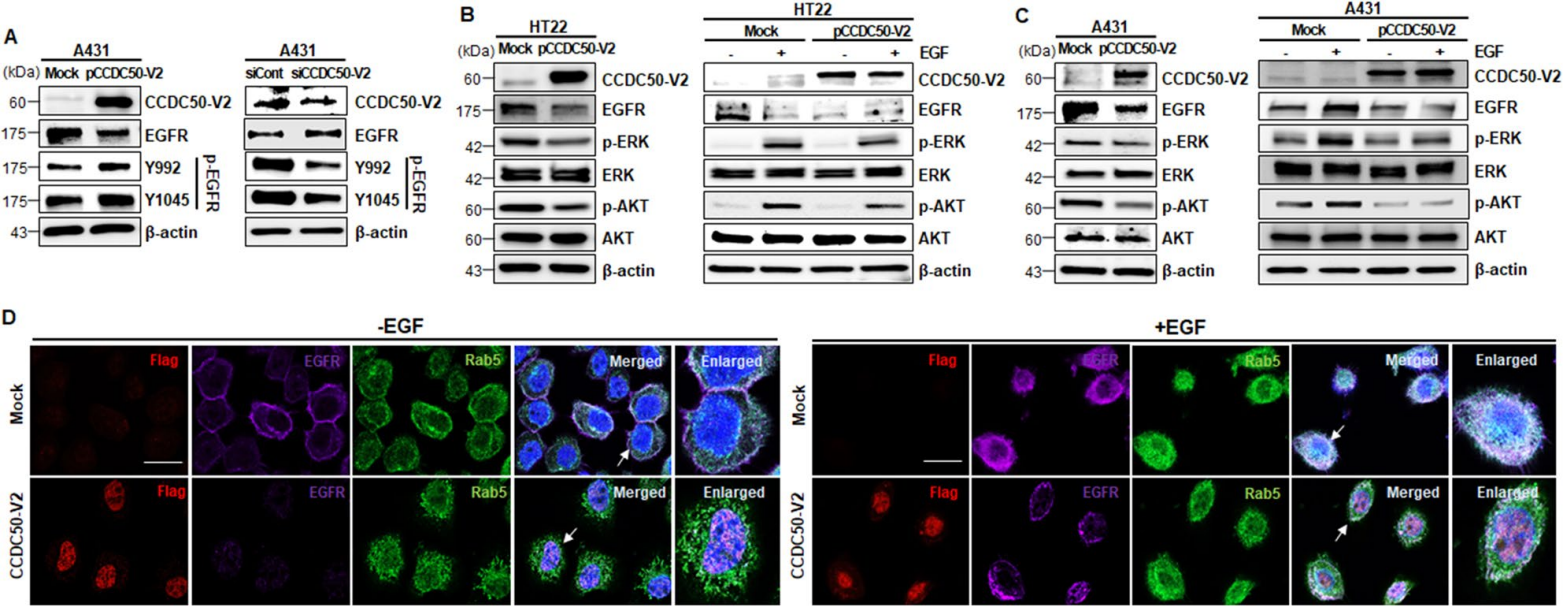
CCDC50-V2 negatively regulates the EGFR signalling pathway. (**A**) The levels of EGFR and p-EGFR in *CCDC50-V2*-overexpressing or -knockdown A431 cells. A431 cells were transfected with mock (Flag-tagged control vector), pCCDC50-V2, siCont, or siCCDC50-V2 and then immunoblotted with the indicated antibodies. (**B**,**C**) The phosphorylation of ERK and AKT, which are downstream of EGFR, was decreased upon the overexpression of *CCDC50-V2*. After 18 h serum starvation, mock- and pCCDC50-V2-transfected cells were incubated with or without EGF (50 ng/ml for 15 min in HT22 cells and 50 ng/ml for 5 min in A431 cells) and then cells were subjected to immunoblotting with the indicated antibodies. (**D**) Immunocytochemistry of A431 cells with or without EGF stimulation. Transfected cells were stained with the indicated antibodies, such as anti-Flag (red, CCDC50-V2), anti-EGFR (Far red, EGFR), anti-Rab5 (green, endosomal marker; Rab5), and DAPI (blue, nuclei). The right lanes contain magnifications of the arrow indicated cells in the merged images. Scale bars = 40 µm.

Previous studies have shown that CCDC50 activates the EGF signalling pathway through the inhibition of EGFR ubiquitination in A431 cells^2,10^. To gain insights into the function of CCDC50 in neuronal development, we investigated the role of CCDC50 through its association with the EGF signalling pathway. The results of the western blot analysis revealed that the overexpression of *CCDC50-V2* led to a decrease in EGFR expression and increased phosphorylation of EGFR-Y1045, which is a phenomenon that has been reported in EGFR degradation by ubiquitination^18–20^, suggesting the activation of EGFR degradation in CCDC50-V2-overexpressing cells (Fig. 3A; left panel). Moreover, we observed that the reduced expression level of EGFR was dependent on the expression level of *CCDC50-V2* (Supplementary Fig. S2A). In line with these observations, immunocytochemical analysis revealed that the overexpression of *CCDC50-V2* resulted in a decrease in EGFR expression and an increase in p-EGFR-Y1045 levels (Supplementary Fig. S2C). In contrast, the knockdown of *CCDC50-V2* led to an increase in EGFR expression and a decrease in p-EGFR-Y1045 levels (Fig. 3A; right panel). Further support for the negative effect of CCDC50-V2 on EGFR downregulation was found in the observation that CCDC50-V2 was overexpressed in the HT22 mouse neuronal cell line (Fig. 3B).

The phosphorylation of EGFR-Y992 is known to negatively regulate the Ras-ERK signalling cascade induced by EGF^11,21^. Because of the increase in EGFR-Y992 in CCDC50-V2-expressing cells (Fig. 3A and Supplementary Fig. S2C), we examined the status of ERK and protein kinase B (AKT) in these cells. As expected, decreases in the levels of phosphorylated ERK and AKT were observed in *CCDC50-V2*-overexpressing HT22 and A431 cells compared to control cells (Fig. 3B,C; left panels), which further indicates the inactivation of EGFR activity and its downstream signalling events. Moreover, this phenomenon was observed even in *CCDC50-V2*-overexpressing HT22 and A431cells upon EGF stimulation. It was revealed that the stimulation of mock control cells with EGF increased the expression of phosphorylated ERK and AKT, while CCDC50-V2 overexpression inhibited the EGF-induced expression of EGFR targets (Fig. 3B,C; right panels). In contrast to its overexpression, the silencing of *CCDC50-V2* led to a concomitant decrease in p-EGFR-Y992 and an increase in p-ERK upon EGF stimulation (Fig. 3A; right panel, and Supplementary Fig. S2D).

In general, EGFR signalling occurs at the plasma membrane. Upon binding of ligands such as EGF to EGFRs, the activated EGFRs then translocate to the cytosol through endosomal vesicle formation^22^. We next examined endosomal vesicle formation in both *CCDC50-V2*-overexpressing and control cells upon EGF stimulation. As expected, in control cells, EGF treatment induced the formation of endosomal vesicles and the translocation of the EGF-EGFR complex, as observed by the colocalization of EGFR and Rab5, which is an endosomal marker (Fig. 3D and Supplementary Fig. S3). However, these observed changes were less prominent in *CCDC50-V2-*overexpressing cells. Overall, these data provide strong experimental evidence that *CCDC50-V2* inhibits the EGF/EGFR signalling pathways.

### CCDC50-V2 positively regulates the NGFR signalling pathway

**Figure 4:**
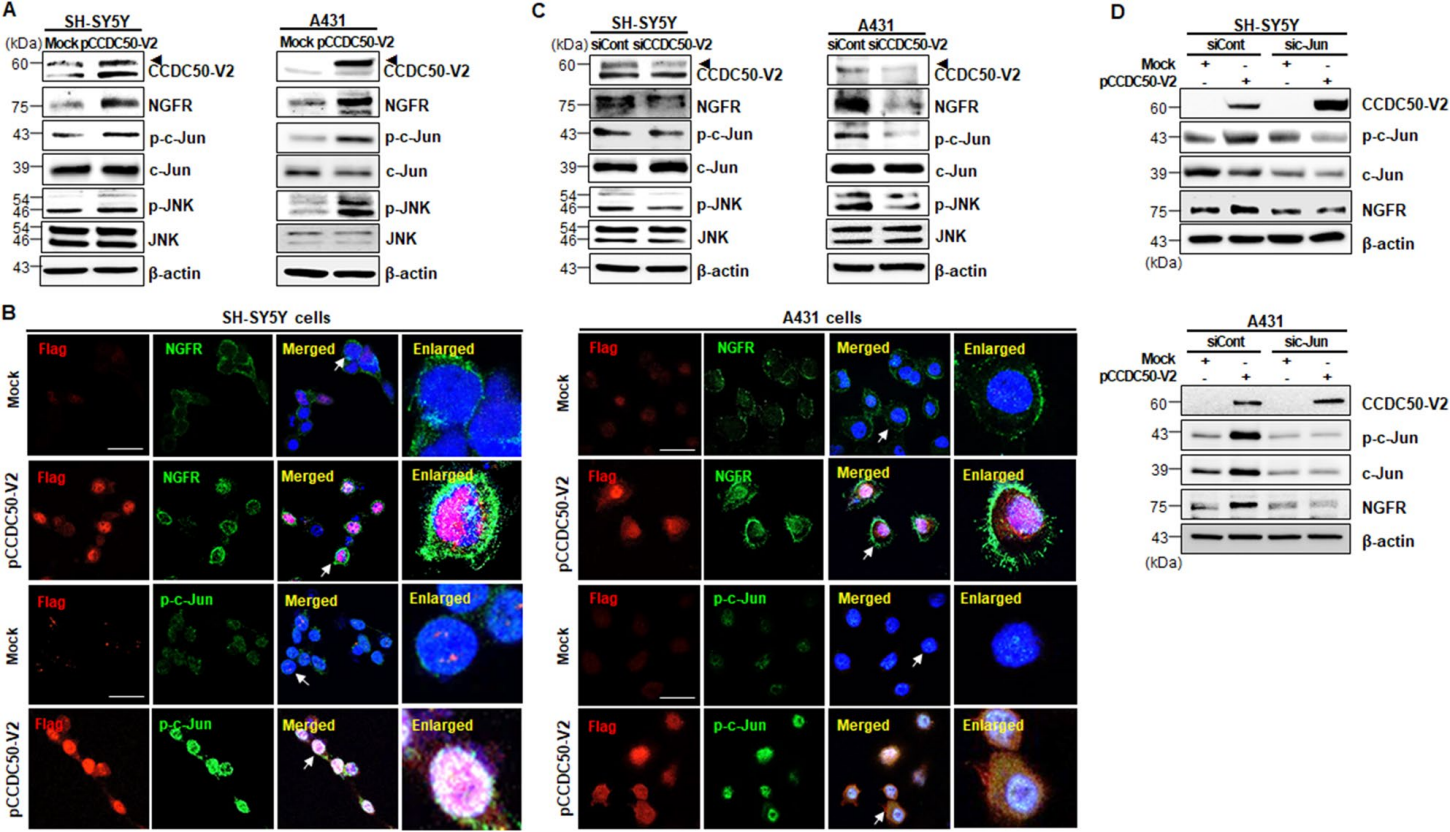
CCDC50-V2 activates the NGFR signalling pathway. (**A**–**C**) The levels of p-JNK/p–c-Jun/NGFR in *CCDC50-V2*-overexpressing (**A**,**B**) or knockdown (**C**) SH-SY5Y and A431 cells. SH-SY5Y and A431 cells were transfected with mock, pCCDC50-V2, siCont, or siCCDC50-V2 and then immunoblotted with the indicated antibodies. (**B**) *CCDC50-V2*-transfected cells were stained with an anti-Flag (red, CCDC50-V2), anti-NGFR (green, NGFR), or anti-p–c-Jun antibody (green, p–c-Jun) and with DAPI (blue, nuclei). Scale bars = 40 µm. (**D**) SH-SY5Y and A431 cells were transfected with mock, pCCDC50-V2, siCont, or sic-Jun and then immunoblotted with the indicated antibodies. CCDC50-V2-mediated NGFR upregulation was suppressed by the co-transfection of c-Jun specific siRNA.

EGF signalling and NGF signalling are known to share the same pathway to promote distinct biological functions. Previous studies have suggested that EGFR plays a role as an activator of cell proliferation, while the stimulation of NGFR leads to cell differentiation by activating a growth factor/receptor pathway in neuronal cells^23^. NGFR is also known to function as an important regulator of the survival of neurons^24,25^. Based on these reports, we evaluated NGFR expression in SH-SY5Y and A431 cells overexpressing CCDC50-V2. Immunoblotting data revealed an increase in NGFR expression in CCDC50-V2-overexpressing cells compared to control cells (Fig. 4A). These findings were further confirmed by immunocytochemical analysis of CCDC50-V2-overexpressing SH-SY5Y and A431 cells (Fig. 4B). Conversely, a decrease in NGFR expression was detected in *CCDC50-V2*-silenced SH-SY5Y and A431 cells (Fig. 4C). Together, these results suggest a correlation between NGFR expression and neurite outgrowth by perturbation of CCDC50.

To gain mechanistic insight into the role of NGFR in increase of neurite outgrowth in *CCDC50-V2*-expressing cells, we investigated the effects of *CCDC50-V2* expression on the MAPK pathway, which is downstream of NGFR signalling. We observed that, unlike the phosphorylation of ERK, the phosphorylation of JNK and c-Jun was increased in *CCDC50-V2*-overexpressing cells compared to control cells (Fig. 4A,B). However, this induced expression of phosphorylated JNK and c-Jun was abolished by CCDC50-V2 depletion in both SH-SY5Y cells and A431 cells (Fig. 4C). In addition, c-Jun depletion suppressed the activation of c-Jun and the increase in NGFR expression in CCDC50-V2-overexpressing SH-SY5Y cells and A431 cells (Fig. 4D). A JNK/c-Jun-mediated increase in NGFR expression was also observed in HT22 cells overexpressing CCDC50-V2 (Supplementary Fig. S2B). Interestingly, an increase in p-JNK was also observed in the cerebral cortices of mice on postnatal day 1, when neuronal development occurs, and was concomitant with high expression of CCDC50-Vx3, MAP2 and TUBB3 (Supplementary Fig. S1C). Taken together, these results suggest that CCDC50-V2 increases the expression of NGFR and several neuronal factors, which might induced the neuronal development process.

### CCDC50-V2 interacts with JNK

**Figure 5:**
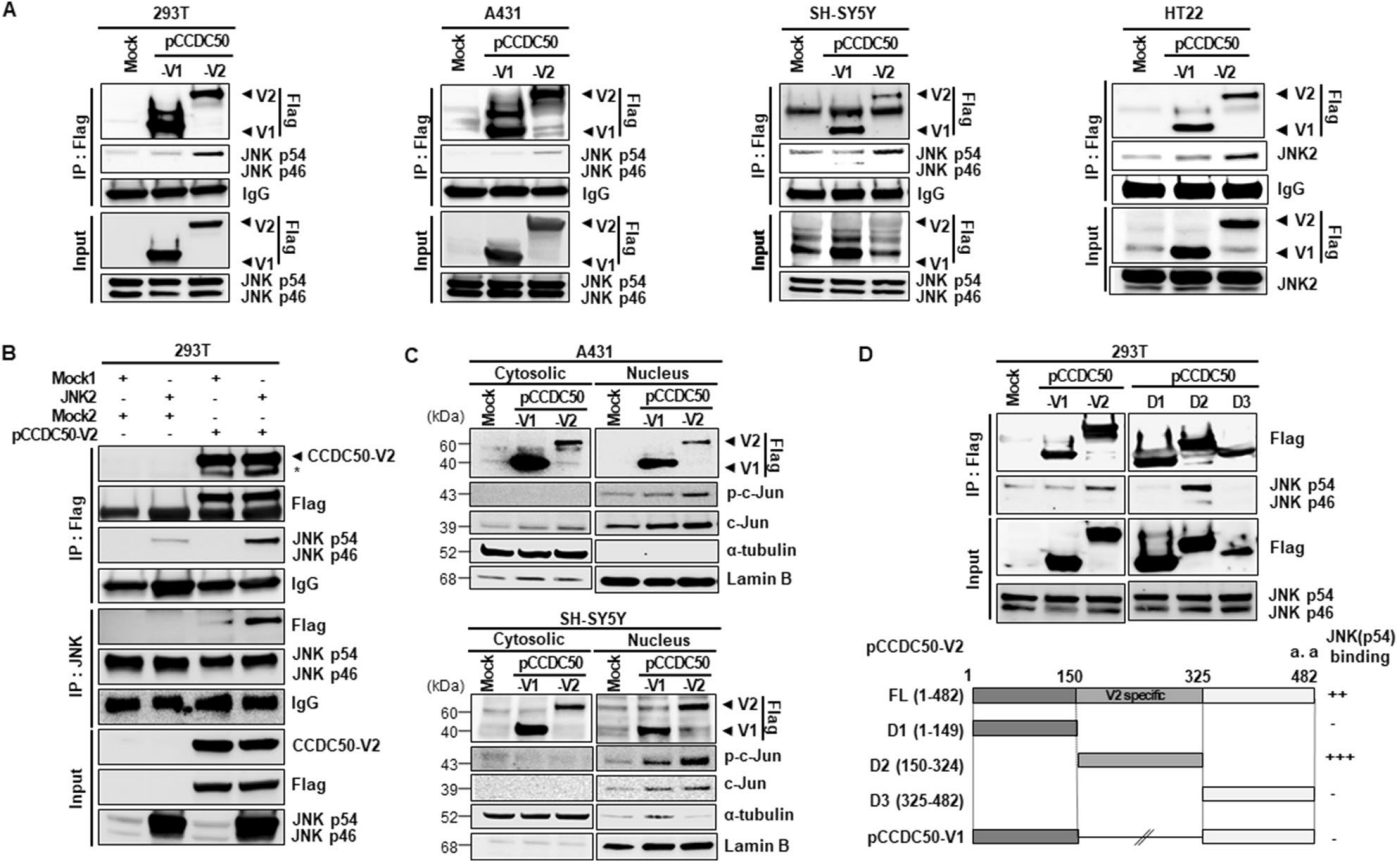
An interaction between CCDC50-V2 and JNK in cells. (**A**) The interaction between CCDC50-V2 and JNK was detected in 293 T, A431, SH-SY5Y and HT22 cells by immunoprecipitation (IP) using anti-Flag-tagged beads, and the pellets were analysed by immunoblotting with the indicated antibodies. (**B**) The specific interaction between CCDC50-V2 and JNK2 was confirmed in 293 T cells co-transfected with JNK2 and CCDC50-V2 vectors by reverse IP using anti-Flag-tagged beads and an anti-JNK antibody with agarose beads. (**C**) CCDC50-V2 overexpression results in increased translocation of p–c-Jun from the cytosol to the nucleus. A431 and SH-SY5Y cells were transfected with the indicated vectors (0.5 μg/ml), harvested at 24 h, and separated into cytoplasmic and nuclear fractions. The blots were cropped from different parts of the same blot and full length blots are presented in Supplementary Figure S6. (**D**) To determine the JNK-specific binding region of CCDC50-V2, various deletion fragments of *CCDC50-V2* were cloned into the pCMV6-entry vector (deletion constructs: 1–149 [D1], 150–324 [D2] and 325–482 [D3]). The protein–protein interactions between the V2-specific regions of CCDC50-V2 (D2) and JNK were investigated using deletion constructs in 293 T cells.

To address the direct impact of JNK on CCDC50-V2-mediated neuronal outgrowth, 293T, A431, SH-SY5Y and HT22 cells transfected with a CCDC50-V1 or CCDC50-V2 construct were assessed using immunoprecipitation (IP). The IP data showed that CCDC50-V2 interacted strongly with endogenous JNK, particularly the 54-kDa JNK isoform 2 (JNK2), while CCDC50-V1 did not bind it (Fig. 5A). In addition, the binding of JNK2 to CCDC50-V2 was confirmed in cells transfected with *JNK2* and *CCDC50-V2* (Fig. 5B). The interaction was also observed by dot-blot overlay assay (Supplementary Fig. S4). The main cellular substrate mediated by activated JNK is c-Jun, which in turn is able to activate the transcription of target genes in the nucleus. We observed that CCDC50-V2 overexpression results in increased translocation of p–c-Jun from the cytosol to the nucleus and increased accumulation of this molecule (Fig. 5C), indicating that p–c-Jun functions as a transcription factor that activates NGFR expression. However, CCDC50-V2 did not interact with the JNK substrate c-Jun or with MAPK kinase 4/7 (MKK4/7), upstream kinases of JNK (Supplementary Fig. S5A,B). These findings indicate that CCDC50-V2 interacts with JNK2, a downstream regulator of NGFR signalling.

It was previously revealed that CCDC50 consists of one coiled-coil domain and two motifs that interact with ubiquitin domains that are known to function as a binding site for the ubiquitinated EGF receptor^2,3^. Based on this, we generated a series of deletion constructs of CCDC50-V2, such as D1 (amino acids 1–149), D2 (amino acids 150–324), and D3 (amino acids 325–482), to determine the region of CCDC50-V2 that binds with JNK2. As demonstrated in Fig. 5D, only the D2 construct of CCDC50-V2, exon 6 region, interacted with endogenous JNK2. These results were in accordance with IP data that showed specific binding of JNK2 to CCDC50-V2 but not to CCDC50-V1. These results suggest that CCDC50-V2 interacts with JNK2 through the specific region of CCDC50-V2.

## Discussion

**Figure 6:**
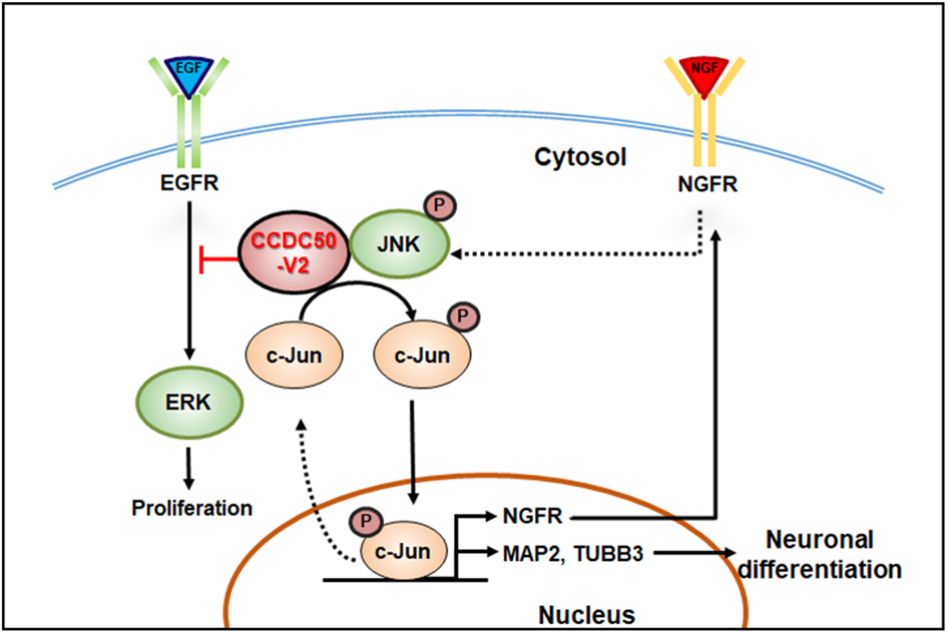
Schematic representation of the proposed mechanism of the involvement of CCDC50-V2 in neuronal development.

In this study, we show that CCDC50-V2 is associated with the development of neuronal phenotypes. The loss of function of CCDC50-V2 resulted in a decrease of axon and dendrite outgrowth, indicating that CCDC50-V2 plays a crucial role in establishing the axodendritic architecture in neuron cells through the receptor-mediated pathway (Fig. 6). In addition, our data revealed that CCDC50-V2 induces the activation of JNK/c-Jun and upregulation of NGFR signalling, which might be occurs through an interaction between CCDC50-V2 and JNK2.

CCDC50 has been identified as a tyrosine-phosphorylated protein, and the tyrosine phosphorylation of CCDC50 inhibits the downregulation of EGFR^1–3^. CCDC50 is also involved in the regulation of RTK-mediated signalling pathways and acts as a multifunctional regulator of the large RTK family and various ligands^2,3,26–28^; CCDC50 is therefore presumed to be closely related to growth factor signalling. Here, we observed that CCDC50-V2 inhibits the EGFR signalling pathway by downregulating the EGFR level and suppressing the ERK and AKT signalling cascade via EGFR phosphorylation. However, surprisingly, this phenomenon was not observed for other variants of CCDC50 (e.g., CCDC50-V1) (data not shown). We also demonstrated that the two variants of *CCDC50* were differentially expressed during the cortical neuronal development process in mice^29^. Unlike that of *Ccdc50-V1*, the expression of *Ccdc50-Vx3* was augmented in cultured primary neurons from mice at DIV 1–5 (Fig. 2C). Moreover, our data revealed that *CCDC50-V2* (*Ccdc50-Vx3* in mouse) has more profound effects than *CCDC50-V1* on neuronal differentiation in SH-SY5Y cells. *CCDC50-V2* silencing inhibited neurite outgrowth in the cells, but *CCDC50-V1* silencing increased neurite outgrowth, indicating the opposite effects of the two variants. These findings suggest that there might be a certain mechanism fine-tuning the balance of the variant levels of CCDC50 for proper development of the nervous system. In many cases, variants are known to perform different functions due to post-transcriptional modification of the primary transcript^30^. Previously, the specific expression patterns of two variants of *CCDC50* were identified in hepatocellular carcinoma cells; *CCDC50-V1* mRNA expression was shown to be highly increased by alternative splicing (exon skipping)^31^. From these results, we surmised that the two variants might exhibit functional differences in the regulation of dendritic and axonal outgrowth in neuronal cells and that CCDC50-V2 might play a more critical role than CCDC50-V1 in the process. Further studies on the control of the spatiotemporal expression of the two CCDC50 variants may provide a better understanding of their fine tuning during nervous system development.

In subsequent functional studies, we demonstrated that CCDC50 regulated not only neurite length but also the number of primary and secondary dendrites as well as dendritic arborization. The knockdown of *CCDC50-V2* significantly reduced the mRNA expression level of neuron-specific cytoskeletal markers such as MAP2 and TUBB3. MAP2 and TUBB3 are involved in microtubule assembly and play a role in determining and stabilizing axonal and dendritic shapes during neuronal development. They are accordingly observed within the axodendritic region of mature neurons^32,33^. Thus, the inhibition of neuronal markers and axodendritic architecture disruption in CCDC50-V2-silenced cells further indicates that CCDC50-V2 plays a crucial role in neuronal development. In addition, we observed that the activation of p-JNK and p–c-Jun activity is associated with increased expression of CCDC50-V2. The activation of JNK has been shown to play a crucial role in various pathological and physiological processes, such as cancer and neuronal development (including brain morphogenesis and axodendritic architecture)^34–38^. Moreover, c-Jun is a well-known transcription factor required for the regeneration of axons in injured neurons; it aids in the functional recovery of damaged neurons^39^. Therefore, these findings suggest that CCDC50-V2 induces neuronal characteristics such as neuronal outgrowth, in which the activated JNK/c-Jun pathway might be played an important role.

Furthermore, we uncovered that CCDC50-V2 binds to endogenous JNK (54 kDa) but fails to bind to its substrate c-Jun and upstream kinases, such as MKK4 and MKK7. The truncated mutant study clearly indicated that the V2-specific region containing the amino acids 150–324 has a specific binding site for JNK2. These data suggest that the CCDC50-V2-JNK2 interaction might be linked to JNK activation by CCDC50-V2, with the V2-specific region playing a critical role in the formation of CCDC50-V2 and JNK2 complexes. As we demonstrated that the activation of JNK by CCDC50-V2 is concomitant with the appearance of phosphorylated c-Jun in the nucleus, it is likely that CCDC50-V2 regulates the transcription of genes dependent on c-Jun activity. However, more work on the roles of CCDC50-V2 in the JNK/c-Jun pathway under in vivo conditions needs to be carried out to elucidate the involvement of this pathway in neuronal developmental process.

In reference to neuronal outgrowth caused by *CCDC50-V2* expression, upregulation of NGFR was observed with a concomitant downregulation of EGFR. EGF stimulation produces transient activation of MAPK, leading to cell proliferation, while NGF initiates sustained MAPK activation and induces cell differentiation^40^. In addition, before cells commit to neuronal differentiation and neurite outgrowth by NGFR activation, it is typically required that they withdraw from the cell cycle to stop their proliferation. This is considered the fundamental event at the beginning of neuronal cell development^31,41,42^. In our study, we ascertained that the expression of *CCDC50-V2* caused an increase in NGFR and the phosphorylation of JNK/c-Jun, while it decreased EGFR and ERK phosphorylation. These findings suggest that CCDC50-V2 plays a crucial role in the terminal differentiation of cells into the desired neuronal phenotypes that coincides with proliferation arrest through the regulation of tyrosine receptor kinases such as EGFR and NGFR.

Overall, in this study, we described a new molecular mechanism of CCDC50-V2 function, showing that it is involved in the control of neurite outgrowth, which might be through the positive regulation of NGFR activity and its downstream signalling events. Our findings also provide evidence that CCDC50-V2 negatively regulates the EGFR pathway to play a crucial role in cell fate control during neural development. Finally, the novel molecular mechanism demonstrated in this study will help us understand axodendritic architecture during neuronal development.

## Methods

### DNA constructs

A wild-type human *CCDC50-V2* vector was purchased from OriGENE (Rockville, MD, USA; RC216523). This vector was used as a template to generate truncated mutants of *CCDC50* (Supplementary Table 1). The mutations were confirmed by DNA sequencing (Bioneer, Daejeon, Korea). A wild-type human *JNK2* vector was provided by the Korea Human Gene Bank (Medical Genomics Research Center, KRIBB, Korea). For knockdown of *CCDC50* (human, NM_174908.3 and NM_178335.2; mouse, XM_011246002.2; rat, NM_182736.1), CCDC50-shRNA was produced by sub-cloning targeted nucleotides into pSuper.gfp/neo (OligoEngine, Seattle, WA, USA).

### Cell culture and differentiation

Human skin cancer A431 cells, human embryonic kidney HEK293T (293 T) cells and mouse neuronal HT22 cells were grown in Dulbecco’s modified Eagle’s medium (DMEM)/high glucose with L-glutamine and sodium pyruvate (HyClone, Logan, UT, USA; SH30243) supplemented with 10% foetal bovine serum (FBS; Gibco, Gaithersburg, MD, USA; #16000) and 1% penicillin/streptomycin. Human neuroblastoma SH-SY5Y cells were grown in DMEM/high glucose with sodium pyruvate without L-glutamine (HyClone; 30285), supplemented with 1% non-essential amino acid (NEAA; Gibco; 11140-050), 1% GlutaMAX supplement (Gibco; 35050-061), 1% penicillin/streptomycin (Gibco; 15140), and 10% FBS. The cells were maintained in 5% CO_2_ in a 37 °C incubator. The neuronal differentiation method for SH-SY5Y cells was used as described previously with some modifications^42^. The differentiation of SH-SY5Y cells was carried out in two steps using retinoic acid (RA; Sigma-Aldrich, St. Louis, MO, USA; R2625) and brain-derived neurotrophic factor (BDNF; Sigma-Aldrich; SRP3014). The RA medium consisted of DMEM/high glucose with sodium pyruvate without L-glutamine, 1% NEAA, 1% GlutaMAX supplement, 1% penicillin/streptomycin, and 3% FBS. The medium was further supplemented with all-trans RA (10 μM) before it was applied to the cells. After 24 h of RA treatment, half of the medium was replaced with fresh medium containing RA. After 120 h of RA treatment, BDNF medium containing DMEM/high glucose with sodium pyruvate without L-glutamine, 1% NEAA, 1% GlutaMAX supplement, 1% penicillin/streptomycin but without serum was added. Fresh BDNF (50 ng/ml) was added shortly before applying the medium to the cells.

### Primary neuronal culture

Primary hippocampal and cortical neurons were prepared as previously described^43^. Hippocampi and cerebral cortices were dissected from the brains of embryonic day 14 (E14) C57Bl/6 mice or embryonic day 18 (E18) rats and dissociated physically using Pasteur pipettes after trypsin treatment. Plating medium (DMEM/F-12 (Gibco; 11330-032), N-2 supplement (Gibco; 17502048), and NEAA) was used to plate the primary neuronal cells. The cells were maintained in neurobasal medium (Gibco; 21103049) with B-27 supplement (Gibco; 17504044) and 1% GlutaMAX supplement (Gibco; 35050). On day in vitro 3 (DIV 3), mouse neurons were grown on poly-D-lysine (Sigma; P7280; 10 μg/ml)-coated glass coverslips and transfected with vector or small interfering RNA (siRNA) using Lipofectamine 2000 (Invitrogen; 11668) or RNAiMAX (Invitrogen; 13778). On DIV 6, the mouse neurons were used for the indicated experiments. Rat neurons were grown on glass coverslips on DIV 5 and transfected using the calcium-phosphate method.

### RNA isolation and PCR analysis

For polymerase chain reaction (PCR), cells were seeded in a plate (293 T and A431 cells: 1 × 10^5^ cells/well in a 6-well plate; primary cultured neurons: 1 × 10^6^ cells/60 mm^2^ dish). After 36 h of incubation, siRNA transfection was conducted using Lipofectamine RNAiMAX. The concentration of siRNA was 100 nM. The transfected cells were allowed to grow for another 36 h. Afterwards, the cells were harvested, RNA was extracted using an RNeasy Mini Kit (Qiagen, Valencia, CA, USA), and 1 μg of RNA was reverse-transcribed using an iScript cDNA synthesis kit (Bio-Rad, Hercules, CA, USA). Reverse-transcription PCR (RT-PCR) was performed using the AccuPower PCR Premix (Bioneer). Quantitative real-time PCR was performed using the Bio-Rad PCR system (iQ SYBRR Green Supermix). The reactions were performed in triplicate. The PCR primers used in this study are described in Supplementary Table 1.

### Protein complex immunoprecipitation

For protein complex immunoprecipitation (co-IP), cells were seeded in 100-mm^2^ dishes (1 × 10^6^/well). After 36 h, transfections were performed using Lipofectamine 2000 (Invitrogen; 11668) according to the manufacturer’s protocol. The transfected cells were allowed to grow for another 36 h before the cells were lysed with 600 µl of immunoprecipitation (IP) lysis buffer containing a protease and phosphatase inhibitor cocktail (Thermo Fisher Scientific, Waltham, MA, USA; 87787) and centrifuged at 13,000×*g* for 10 min. The supernatant was incubated with 50 µl of anti-FLAG M2 affinity gel (Sigma; A2220) or protein G-agarose (Roche, IN, USA; 11719416001) for 4 h. The beads were washed five times with 1 × Tris-buffered saline buffer. The proteins were eluted with Laemmli sample buffer (Bio-Rad; #161-0747) and boiled at 95 °C for 20 min. A quarter of the total eluate was resolved using SDS-PAGE with precast protein gels (Bio-Rad; #456-1094).

### Immunoblotting

For immunoblotting, protein samples were prepared by cell lysis with radioimmunoprecipitation assay buffer (150 mM NaCl, 20 mM Tris–HCl pH 7.4, 2 mM NaF, 2 mM EDTA, 5 mM sodium orthovanadate, 1% Triton X-100, 1 mM phenylmethylsulfonyl fluoride, and protease inhibitor cocktail) or IP sample buffer. The protein samples were mixed with Laemmli sample buffer and boiled at 95 °C for 20 min. They were separated using SDS-PAGE (10–15 mA/gel) with precast protein gels. The separated proteins were transferred to a 0.45-μm nitrocellulose membrane (GE Healthcare Life Sciences, Issaquah, WA, USA) and blocked in 5% skim milk in Tris-buffered saline with Tween 20 for 1 h at room temperature. The membrane was incubated with primary antibodies in Can Get Signal (Toyobo, Osaka, Japan; NKB-101) solution 1 overnight at 4 °C and in secondary antibodies in solution 2 for 1 h at room temperature.

### Dot blot overlay assay

Two-fold serial dilutions of purified GST-JNK1 and His-JNK2 proteins (Sino Biological Inc.) in binding buffer [20 mM HEPES (pH 7.4), 0.1 mM EDTA, 150 mM KCl, and 0.1% Triton-X100 (v/v)] were spotted onto a nitrocellulose membrane. Dilution of BSA controls were the same as the GST and His tagged proteins. The membrane strip was then blocked using 5% skim milk for 1 h and then was incubated overnight at 4 °C with 300 μg of 293 T cell lysates expressing mock or GFP-CCDC50-V2. The membrane was incubated overnight at 4 °C with an anti-GFP antibody (1:1000) in 1% skim milk binding solution and with a secondary antibody (1:2000) for 1 h at room temperature.

### Transfection and immunocytochemistry

For immunocytochemistry, cells were grown on poly-D-lysine-coated glass coverslips. Constructed vectors and siRNA were transfected into A431, SH-SY5Y, HT22, and 293 T cells using Lipofectamine 2000 or RNAiMax. After transfection, A431 and HT22 cells were serum-starved for 18 h and then stimulated with EGF (Sigma; E9644) or nerve growth factor (NGF; PeproTech, Rocky Hill, NJ, USA; 405-01) for the indicated time. The cells were washed once in phosphate-buffered saline (PBS) and fixed in 4% paraformaldehyde (pH 7.4) for 10 min. The fixed cells were blocked in 5% bovine serum albumin for 30 min. The fixed and permeabilized cells were incubated with the indicated primary antibodies (500:1) for 12 h at 4 °C. After the primary antibodies were removed by washing the cells twice with PBS, the cells were incubated with Alexa Fluor-conjugated secondary antibodies (1000:1). The nuclei were stained with 4′, 6-diamidino-2-phenylindole dihydrochloride (DAPI; Vector Laboratories; Burlingame, CA, USA; H-1200).

### Image analysis and quantification

Neurite outgrowth was analysed using a fluorescence microscope (Nikon Eclipse Ti-s; Nikon, Tochigi, Japan) and NIS-Elements software (Nikon, version 4.3). Quantification of the neurite length of primary neurons and differentiated SH-SY5Y cells was also determined from images of individual neurons taken using a 10 × objective. Sholl analysis was performed using a previously described modified method^43,44^. Individual neurons and fixed cells were imaged using confocal microscopy (LSM 510 Meta and 800; Zeiss, Göttingen, Germany) with a 20 × and 40 × objective, and the images were printed. To obtain the Sholl profiles of dendritic arbors, printouts were placed under a clear sheet featuring concentric circles with diameters that increased in 20 μm increments. The centre of the circles was placed on the cell body centre, and the number of dendrites that crossed each concentric circle was counted. Quantification of primary and secondary dendrites was also obtained from images of individual neurons taken using a 20 × objective.

To analyse dendritic spine density, images were captured using confocal microscopy with a 63 × objective and examined blindly using MetaMorph software (Universal Imaging), as previously described^45^. The density of dendritic protrusions (0.4–2.5 μm) was measured from 70 to 90 dendrites from 14 to 18 neurons; a total dendritic length of ~ 50 μm was measured from the first dendritic branching points. Means from multiple individual dendrites were averaged to obtain a population mean and SD.

### Statistical analysis

All experiments were performed in triplicate. All statistical analyses were performed with GraphPad Prism software (GraphPad Software Incorporated, CA, USA) or Microsoft Excel. The error bars represent the standard deviation (SD). An unpaired, two-tailed Student’s t-test or analysis of variance was used for statistical analyses unless otherwise specified. Differences with *p* values less than 0.05 were considered significant. Significance levels are expressed as follows: *p < 0.05, **p < 0.01, and ***p < 0.001.

## Supplementary information

Supplementary Information.
