# Supplementary material for: Coiled-coil domain containing 50-V2 protein positively regulates neurite outgrowth
Source: Sci Rep. 2020 Dec 4;10:21295. doi: 10.1038/s41598-020-78304-3 (PMC7718278; doi:10.1038/s41598-020-78304-3)
Supplement: Supplementary file 1 — Supplementary Information. [file 41598_2020_78304_MOESM1_ESM.pdf]

Coiled-coil domain containing 50-V2 protein positively regulates neurite outgrowth

Ju-Sik Min[1,2,5,†], Debasish Halder[1,†], Ji-Yong Yoon[1,2], Su-Jin Jeon[1,3], Soo Young Jun[1,2], Jae-Ran Lee[1,3], Jeong-Ju Lee[1,2], Min-Hyuk Choi[1,3,], Cho-Rok Jung[1,3], DaYong Lee[1], Byoung-Joon Kim[4] & Nam-Soon Kim[1,2,3,*]

[1]Rare Disease Research Center, [2]Genome Research Center, Korea Research Institute of Bioscience and Biotechnology, 34141 Daejeon, Republic of Korea; [3]Department of Functional Genomics, Korea University of Science and Technology, 34113 Daejeon, Republic of Korea; [4]Department of Neurology, Sungkyunkwan University School of Medicine, Samsung Medical Center Gangnam-gu, 06351 Seoul, Republic of Korea. [5]New Drug Development Center, Daegu-Gyeongbuk Medical Innovation Foundation, Daegu, Republic of Korea.

[†]*These authors contributed equally to this work.*

[*]**Correspondence:** Nam-Soon Kim

Rare Disease Research Center, Korea Research Institute of Bioscience and Biotechnology

(KRIBB), Daejeon 34141, Republic of Korea

Tel.: +82-42-879-8112, Fax: +82-42-879-8119

E-mail: nskim37@kribb.re.kr

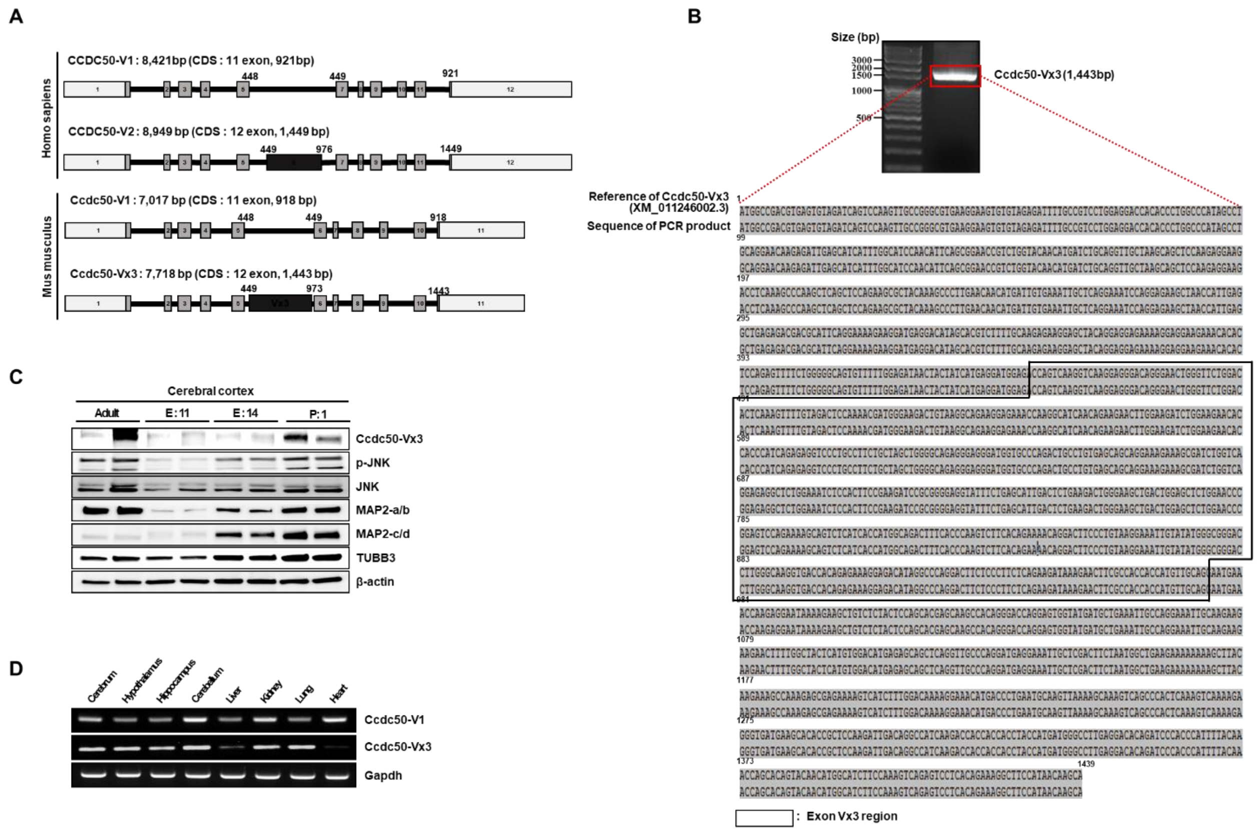

Supplemental Fig. 1. Expression pattern of the Ccdc50 long variant, Ccdc50-Vx3, in mouse tissues and primary neuron cells. **(A)** Schematic representation of the human and mouse *CCDC50* variants. (B) Confirmation of the expression of *Ccdc50-Vx3* by RT-PCR and sequence analysis. **(C)** Expression of mouse *Ccdc50* variants was detected at early three developmental point of mouse. The embryonic 11 day (E11): related to nervous system construction, including positive regulation of neuroblast proliferation. The embryonic 14 day (E14): important stage of brain development including neurogenesis, gliogenesis and neuronal migration. The postnatal day 1(P1): accompany sudden changes including changes of gene expression according to the external environment exposure. **(D)** The mRNA expression pattern of *Ccdc50* variants in mice tissues from C57BL/6J mice (12 weeks aged; male).

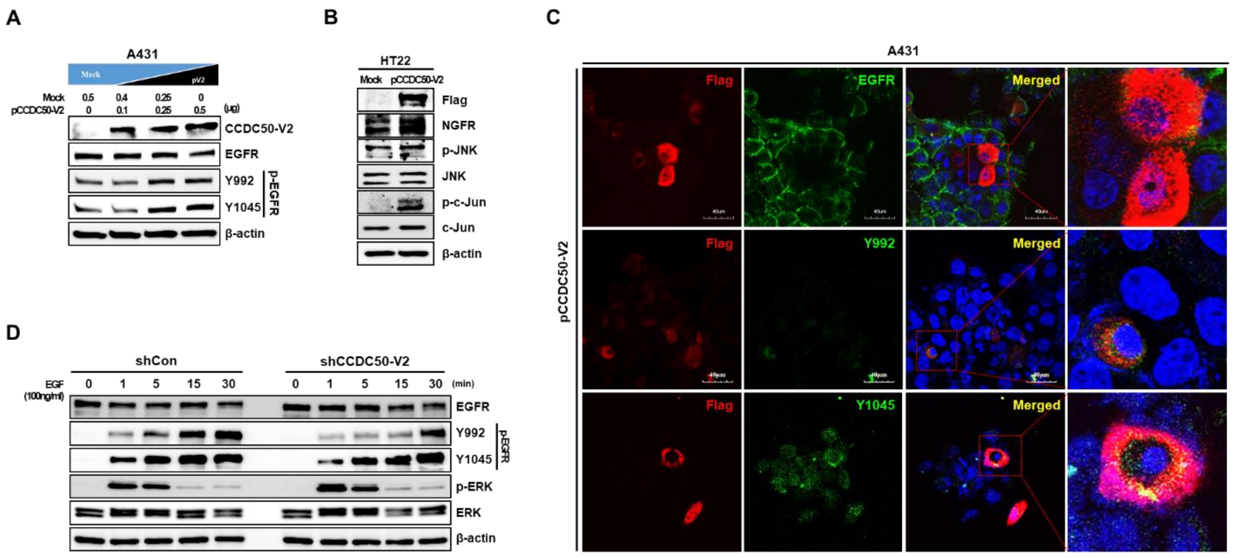

Supplemental Fig. 2. CCDC50-V2 regulates EGFR via phosphorylation of the EGFR and NGFR expression by JNK activation. **(A)** CCDC50-V2 dose dependent EGFR expression, and phosphorylation of EGFR-Y1045 and EGFR-Y992. According to CCDC50-V2 dose, EGFR expression is decreased, and p-EGFR-Y1045 and p-EGFR-Y992 is increased. **(B)** Levels of p-JNK/p-c-Jun/NGFR in *CCDC50-V2*-overexpressing HT22 cells. (C) Immunocytochemical analysis of CCDC50-V2-transfected A431 cells with anti-Flag (red, CCDC50-V2), anti-EGFR antibodies (green, EGFR), and DAPI (blue, nuclei). Scale bars = 40 μm. The right lane contains magnifications of the red-dotted squares in the merged pictures. **(D)** In A431 cell, depletion of *CCDC50-V2* led to a decrease of p-EGFR-Y992 and p-EGFR-Y1045 compared to the control. The suppression of *CCDC50-V2* also caused an increase in EGFR expression and phosphorylation of ERK upon EGF stimulation.

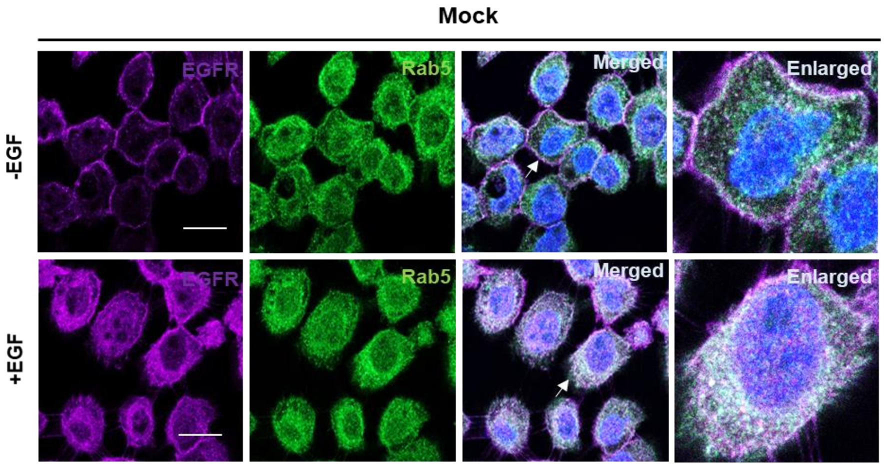

Supplemental Fig. 3. An endosomal localization of EGFR in response to EGF. A431 cells were transfected with mock and then stimulated with or without EGF. The transfected cells were immunostained with the indicated antibodies, such as anti-EGFR (Far red, EGFR), anti-Rab5 (green, endosomal marker; Rab5), and DAPI (blue, nuclei). The right lanes contain magnifications of the arrow indicated cells in the merged images. Scale bars = 40 μm.

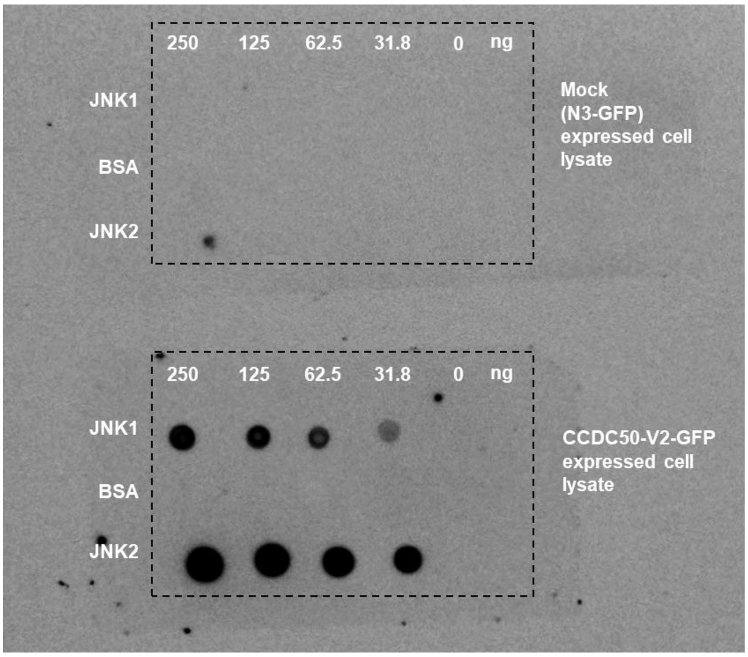

Supplemental Fig. 4. An interaction between CCDC50-V2 and JNK was observed by dot blot overlay assay. Purified GST-JNK1 and His-JNK2 proteins were spotted to a nitrocellulose membrane using double serial dilutions ranging from 250 ng to 0 ng. The membrane was first incubated with the indicated cell lysates, and then with anti-GFP antibodies. The interaction between CCDC50-V2 and JNK1/2 was detected by using chemiluminescence detection system. BSA was used as a negative control.

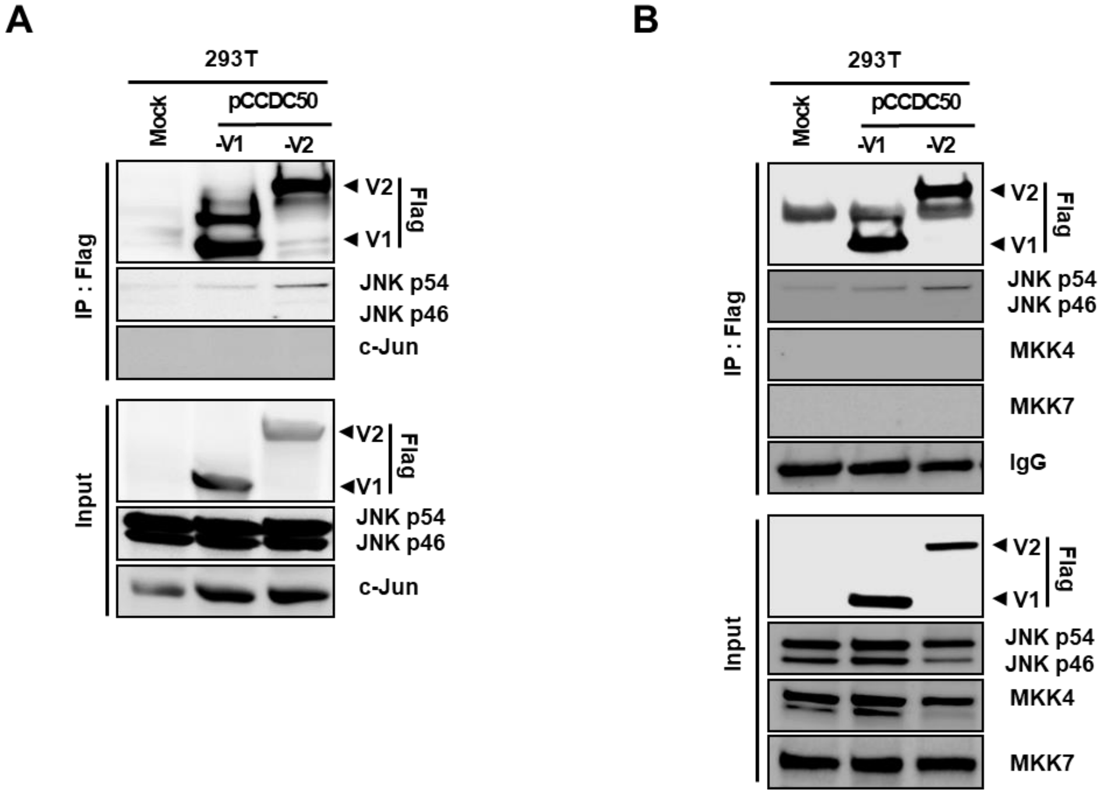

Supplemental Fig. 5. CCDC50-V2 regulates NGFR expression by specific activation of JNK. (A) An interaction between CCDC50-V2 and JNK2 was detected in 293T cells by immunoprecipitation (IP) using anti-flag tagged bead, and the pellets were analyzed by immunoblotting with indicated antibodies. (B) CCDC50-V2 doesn't have binding affinity with c-Jun and MKK4 or 7, substrate and upstream kinase of JNK.

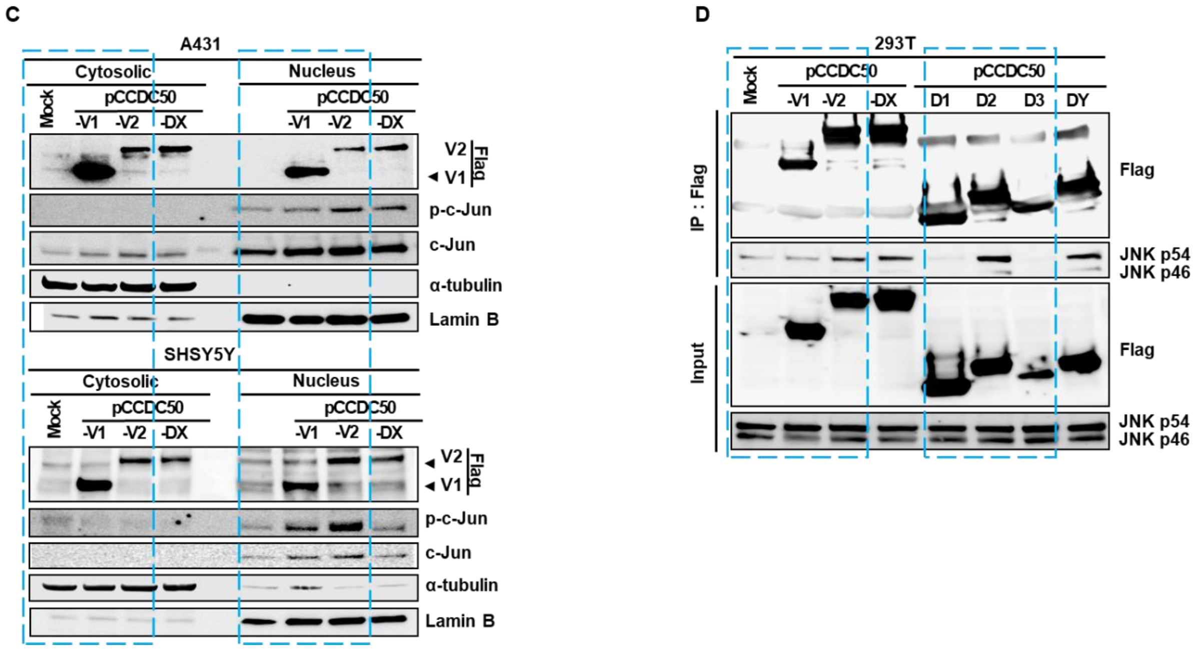

Supplemental Fig. 6. (C) CCDC50-V2 overexpression results in increased translocation of p-c-Jun from the cytosol to the nucleus. A431 and SH-SY5Y cells were transfected with the indicated vectors (0.5 μg/ml), harvested at 24 h, and separated into cytoplasmic and nuclear fractions. (D) To determine the JNK-specific binding region of CCDC50-V2, various deletion fragments of CCDC50-V2 were cloned into the pCMV6-entry vector (deletion constructs: 1–149 [D1], 150–324 [D2], 325–482 [D3]), and DY. The protein-protein interactions between the V2-specific regions of CCDC50-V2 (D2) and JNK were investigated using deletion constructs in 293T cells. Dash lines indicate that portions of cropped blots are added into the main figure (Figure 5C and D).

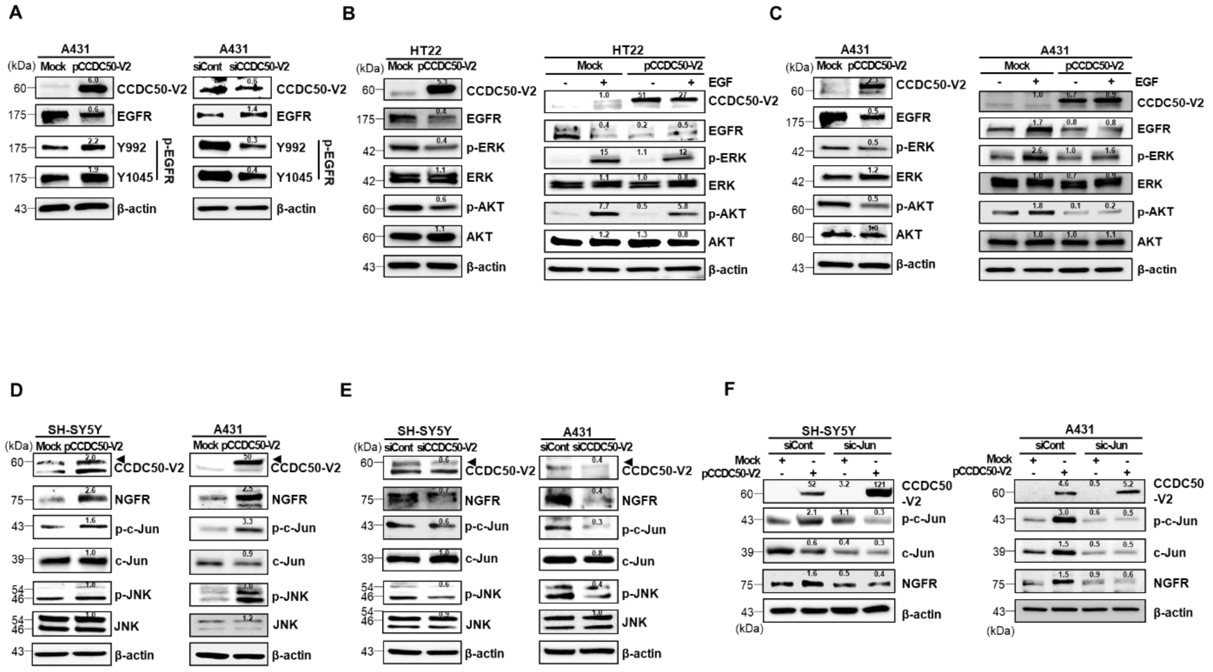

Supplemental Fig. 7. Semiquantitative analysis of the immunoblotting results. (A-C) Images were extracted from Figure 3 and quantified. (D-F) Images were extracted from Figure 4 and measured. For the intensity measurements of each band, we used ImageJ software. The data shown represent the relative amount of proteins in mock or siCont samples, and they are normalized to β-actin levels. The phosphorylation levels of the indicated proteins were calculated relative to the amount of total proteins after normalization against β-actin. The relative amount of p-c-Jun was normalized to β-actin levels (F).

Supplemental Table 1. Key resources

**Reagents or Resources**	**Source**	**Identifier**
**Antibodies**		
CCDC50	Abcam	Cat# ab32884
MAP2	Abcam	Cat# ab11267
TUBB3	Abcam	Cat# ab18207
c-Jun	Santa Cruz Biotechnology, Inc.	Cat# sc-1694
c-Fos	Santa Cruz Biotechnology, Inc.	Cat# sc-52
MKK4	Santa Cruz Biotechnology, Inc.	Cat# sc-7103
β-actin	Santa Cruz Biotechnology, Inc.	Cat# sc-47778
Flag	Sigma-Aldrich	Cat# F1804
Flag	Sigma-Aldrich	Cat# F7425
AKT	Cell Signaling Technology	Cat# 9272
EGFR	Cell Signaling Technology	Cat# 4267
EGFR	R&D system (Biotechne)	Cat#AF231
NGFR/p75NTR	Cell Signalling Technology	Cat# 8238
MKK7	Cell Signaling Technology	Cat# 4172
ERK1/2 (p44/42 MAPK)	Cell Signaling Technology	Cat# 9102
phospho-EGFR(Tyr992)	Cell Signaling Technology	Cat# 2235
phospho-EGFR(Tyr1045)	Cell Signaling Technology	Cat# 2237
phospho-AKT(Ser473)	Cell Signaling Technology	Cat# 9271
phospho-c-Jun(ser63)	Cell Signaling Technology	Cat# 9261
phospho-ERK1/2 (p44/42 MAPK)	Cell Signaling Technology	Cat# 9106

phospho-SAPK/JNK(Thr183/Tyr185)	Cell Signaling Technology	Cat# 9255
SAPK/JNK	Cell Signaling Technology	Cat# 9252
JNK2 (MAPK9)	Flarebio biotech	Cat# p45984
Rab5	Santa Cruz Biotechnology, Inc.	Cat# sc-46692
GFP	Santa Cruz Biotechnology, Inc.	Cat# sc-9996
Goat anti-Mouse lgG-Alexa Fluor 488	Life technologies	Cat# A11001
Goat anti-Mouse lgG-Alexa Fluor 568	Life technologies	Cat# A11004
Goat anti-Rabbit lgG-Alexa Fluor 488	Life technologies	Cat# A11008
Goat anti-Rabbit lgG-Alexa Fluor 568	Life technologies	Cat# A11011
Donkey anti-Goat lgG-Alexa Fluor 680	Life technologies	Cat# A32860
Goat-anti Rabbit IgG-HRP	Santa Cruz Biotechnology, Inc.	Cat# sc-2004
Goat-anti Mouse IgG-HRP	Santa Cruz Biotechnology, Inc.	Cat# sc-2031
Donkey-anti Goat IgG-HRP	Santa Cruz Biotechnology, Inc.	Cat# sc-2033
Normal Mouse IgG	Santa Cruz Biotechnology, Inc.	Cat# sc-2025
Normal Rabbit IgG	Santa Cruz Biotechnology, Inc.	Cat# sc-2027
**Critical Commercial Assays**		
iScript™ cDNA synthesis kit	Bio-rad	Cat# 1708890
AccuPower HotStart PCR PreMix	Bioneer	Cat# K-5051
iQ SYBRR Green Supermix	Bio-rad	Cat# 170-8882
**Experimental Models: Cell Lines**		
A431	KCLB	Cat# 80005
SH-SY5Y	KCLB	Cat# 22266
293T	ATCC	Cat#CRL3216

HT22	-	-
**Organisms/Strains**		
Fetal ICR mouse (DIV 14)	Orient Bio	N/A
**Recombinant DNA**		
CCDC50-V1	Origene	Cat#RC208965
CCDC50-V2	Origene	Cat#RC216523
Truncated CCDC50 D1~D4	This paper	N/A
Human-shCCDC50-V1	This paper	N/A
Human-shCCDC50-V2	This paper	N/A
Mouse-shCCDC50(Pan)	This paper	N/A
Mouse-shCCDC50-Vx3	This paper	N/A
Rat-shCCDC50(Pan)	This paper	N/A
Human JNK2	Korea Human Gene Bank	Cat#hMU005167
GST-JNK1	Sino Biological Inc.	Cat#10795-H09B
His-JNK2	Sino Biological Inc.	Cat#10745-H08B
**Software and Database**		
NIS element software	Nikon (Ver. 4.3)	
MetaMorph software	Universal Imaging	
**Oligonucleotides**		
**siRNA**		
siRNA for universal control<br>Sense :AUGAACGUGAAUUGCUCAATT<br>Antisense :UUGAGCAAUUCACGUUCAUTT	Stpharm	N/A

siRNA for human CCDC50 (Pan)-#1<br>Sense :CUGUCUUCUAUGGUGACUA<br>Antisense :UAGUCACCAUAGAAGACAG	Bioneer	N/A
siRNA for human CCDC50 (Pan)-#2<br>Sense :CACAUGCCUAUCUAGAGAU<br>Antisense :AUCUCUAGAUAGGCAUGUG	Bioneer	N/A
siRNA for human CCDC50-V1<br>Sense :GAAGAUGGAGGAAUGAAGC<br>Antisense :GCUUCAUUCCUCCAUCUUC	Bioneer	N/A
siRNA for human CCDC50-V2 #1<br>Sense :CCCAUAUUAACAAUGAGCAGCAUGA<br>Antisense :UCAUGCUGCUCAUUGUUAAUAU<br>GGG	Stpharm	N/A
siRNA for human CCDC50-V2 #2<br>Sense :UAACAAUGAGCAGCAUGAAAGGAAA<br>Antisense :UUUCCUUUCAUGCUGCUCAUUGU<br>UA	Stpharm	N/A
siRNA for mouse Ccdc50 (Pan) #1<br>Sense :CUGACAUUUUCUGUCAUGU<br>Antisense :ACAUGACAGAAAAUGUCAG	Bioneer	N/A
siRNA for mouse Ccdc50 (Pan) #2<br>Sense :CUCUCAUUUCCAUCCUAGA<br>Antisense :UCUAGGAUGGAAAUGAGAG	Bioneer	N/A
siRNA for mouse Ccdc50-Vx3 #1	Bioneer	N/A

Sense :CAUGGCAGACUUUCACCCAAGUCUU<br>Antisense :AAGACUUGGGUGAAAGUCUGCC<br>AUG		
siRNA for mouse Ccdc50-Vx3 #2<br>Sense :GGACUUCCCUGUAAGGAAAUUGUAU<br>Antisense :AUACAAUUUCCUUACAGGGAAG<br>UCC	Bioneer	N/A
**PCR primer**		
Primers for human CCDC50-V1<br>F:CAAAGACCTTGAACAACAAG<br>R:GGCTTCATTCCTCCATCTTC	Bioneer	N/A
Primers for human CCDC50-V2<br>F:CCCACGATCAGTGGTGAAGT<br>R:AGCCCTGCTTTTTGTGAGGA	Bioneer	N/A
Primers for human GAPDH<br>F:ACATCGCTCAGACACCATG<br>R:ATGACAAGCTTCCCGTTCTC	Bioneer	N/A
Primers for mouse Ccdc50-V1<br>F:CTGAGCTGCTCTCATGTCCA<br>R:CTGAGCTGCTCTCATGTCCA	Bioneer	N/A
Primers for mouse Ccdc50-Vx3<br>F:TCTGGAAGAACACCACCCAT<br>R:GGTTCCAGAGCTCCAGTCAG	Bioneer	N/A
Primers for mouse-Map2	Bioneer	N/A

F:ACCTTCCTCCATCCTCCCTC<br>R:ATTCTTCAGGTCCGGCAGTG		
Primers for mouse-Tubb3<br>F:TCAAGGTAGCCGTGTGTGAC<br>R:GTGGACTCACATGGAGTGGG	Bioneer	N/A
Primers for mouse-Dcx<br>F:AGTTGCTGTGGTTCCACCAA<br>R:GGTCCCCATTGCGGTAGAAA	Bioneer	N/A
Primers for mouse-Gapdh<br>F:AGGCCGGTGCTGAGTATGTC<br>R:TGCCTGCTTCACCACCTTCT	Bioneer	N/A
**Mutation of CCDC50**		
Primers for D1 (1-149)<br>F:GAGGCGATCGCCATGGCTGAAGTCAGCATCGA<br>R:GCGACGCGTTCCATCTTCATAATAGTA	Bioneer	N/A
Primers for D2 (150-325)<br>F:AGGCGATCGCCATGGACCAACCAGGGTCAAGGA<br>R:GCGACGCGTTGCGTCATGGAGGTGGAG	Bioneer	N/A
Primers for D3 (326-482)<br>F:GAGGCGATCGCCATGGCAGGAATGAAGCCAAGA<br>R:GCGACGCGTATGTTTGTAATGAAAACCT	Bioneer	N/A

**shRNA vectors**
Primers for Human-shCCDC50-V1<br>Sense :<br>GATCCCCGAAGATGGAGGAATGAAGCTTCAAGAGAGCTTCATTCCTCCATCTTCTTTTTA<br>Antisense :<br>AGCTTAAAAAGAAGATGGAGGAATGAAGCTCTCTTGAAGCTTCATTCCTCCATCTTCGGG<br>Primers for Human-shCCDC50-V2 #1<br>Sense :<br>GATCCCCGGAAGAGCCAGAACAACATTTCAAGAGAATGTTGTTCTGGCTCTTCCTTTTTA<br>Antisense :<br>AGCTTAAAAAGGAAGAGCCAGAACAACATTCTCTTGAAATGTTGTTCTGGCTCTTCCGGG
Primers for Human-shCCDC50-V2 #2<br>Sense :<br>GATCCCCGAGACTAAGATTAACCATCTTCAAGAGAGATGGTTAATCTTAGTCTCTTTTTA<br>Antisense :<br>AGCTTAAAAAGAGACTAAGATTAACCATCTCTCTTGAAGATGGTTAATCTTAGTCTCGGG
Primers for Rat-shCCDC50 (Pan)<br>Sense :<br>GATCCCCGAAATTGCCCGGAAATTGCTTCAAGAGAGCAATTTCCGGGCAATTTCTTTTTA<br>Antisense :<br>AGCTTAAAAAGAAATTGCCCGGAAATTGCTCTCTTGAAGCAATTTCCGGGCAATTTCGGG
Primers for Mouse-shCCDC50 (Pan)<br>Sense :<br>GATCCCCAGGATGGAGGAATGAAACCTTCAAGAGAGGTTTCATTCCTCCATCCTTTTTTA

Antisense :

AGCTTAAAAAAGGATGGAGGAATGAAACCTCTCTTGAAGGTTTCATTCCTCCATCCTGGG

Primers for Mouse-shCCDC50-Vx3 #1

Sense :

GATCCCCCAGACTTTCACCCAAGTCTTTCAAGAGAAGACTTGGGTGAAAGTCTGTTTTTA

Antisense :

AGCTTAAAAACAGACTTTCACCCAAGTCTTCTCTTGAAAGACTTGGGTGAAAGTCTGGGG

Primers for Mouse-shCCDC50-Vx3 #2

Sense :

GATCCCCCTTCCCTGTAAGGAAATTGTTCAAGAGACAATTTCCTTACAGGGAAGTTTTTA

Antisense :

AGCTTAAAAACTTCCCTGTAAGGAAATTGTCTCTTGAACAATTTCCTTACAGGGAAGGGG
